# Surgical strategy of bilateral synchronous sporadic renal cell carcinoma—experience of a Chinese university hospital

**DOI:** 10.1186/s12957-016-1071-6

**Published:** 2017-02-28

**Authors:** Xiao-Yi Hu, Lei Xu, Jian-Ming Guo, Hang Wang

**Affiliations:** Department of Urology, Zhongshan Hospital, Fudan University, 180 Fenglin Rd, Shanghai, 200032 China

**Keywords:** Kidney neoplasm, Nephron-sparing surgery, Radical nephrectomy, Synchronous, ZS score

## Abstract

**Background:**

The objective of this study is to investigate the optimal therapeutic protocol for BSSRCC.

**Methods:**

A total of 32 BSSRCC patients, including 28 males and 4 females, were enrolled the study from January 2004 to May 2016. The diagnoses were defined by the measurements of CT, ultrasound, and MRI. Patients with hereditary syndrome were excluded. The management of surgical manner, operation staging, and sequence were scheduled in accordance with the tumor’s location and size (based on Zhongshan score, ZS score), as well as the performance status of the patients. Among them, 8 cases were conducted with bilateral surgical procedure simultaneously and 24 cases were implemented with staged operations. NSS on the one side with contralateral RN, and NSS on both sides were performed in 17 and 15 patients separately.

**Results:**

Thirty cases were conducted 56 operations in total. The average operation time was 260 ± 52 min in simultaneous operations and 162 ± 40 min in staged operations. The length of hospital stay in average was 11.5 ± 1.8 and 7.5 ± 1.4 days, respectively. Twenty-eight cases were followed up by 6–138 months. The level of creatinine was elevated in 5 cases without hemodialysis conducted.

**Conclusions:**

The location and size of the carcinomas, and the performance status of patients should be considered in determination of an appropriate surgical approach. Both renal function preservation and tumor eradication were similarly critical, whereas the latter is of more importance. ZS score may be helpful in the dilemma. Longer follow-up period and more patient enrolment are required.

## Background

Bilateral synchronous sporadic renal cell carcinoma (BSSRCC) is an uncommon disease accounting for 3~4.2% renal carcinomas [1, 2]. It is a difficult surgical dilemma for the urologist to formulate the therapeutic strategy for individual patient. Previous study disclosed patients with this type of tumor usually demonstrated a poor prognosis [3]. As usual, bilateral radical nephrectomy (RN) followed by renal replacement was the major management to these patients, which can obtain a 71% 5-year survival in patients undergoing hemodialysis and 86% 5-year survival in patients receiving kidney transplantation [4]. However, increasing evidences in a variety of studies [5–7] suggested the favorable outcomes of the disease recently. Surgical resection may provide the longer beneficial effect of cancer control for patients treated by bilateral RCC, with similar survival between synchronous and metachronous cancers. Meanwhile, the prognosis between the patients with N0M0 synchronous bilateral RCC and the patients with N0M0 unilateral RCC are comparable. Singer et al. [5] reported that the overall survival (OS) was 88% in the patients with synchronous sporadic carcinomas in a median 16-year follow-up period, which indicated the nephron-sparing surgery (NSS) was an essential procedure to bilateral renal tumors. They emphasized that all efforts should be made during operation, so as to protect the renal function. In the present study, we summarize the experience in the treatment of the BSSRCC and suggest the selection criteria of the surgical manner.

## Methods

### Patients’ characteristics

A total of 32 BSSRCC patients, including 28 males and 4 females at Zhongshan Hospital, Fudan University, were enrolled in the study between January 2004 and May 2016. The study was a prospective design, and the data were collected into an institutional review board-approved database. The patients with bilateral metachronous renal tumors, cystic renal masses, familial renal cell carcinoma syndromes, urothelial cell carcinomas, and defined hereditary syndromes were excluded in accordance with the diagnosis criteria [8]. ECOG PS of all the patients were 0~2.

Among all patients, 22 cases were clued unexpectedly in an annual routine physical examination, 5 cases were diagnosed with major complaint of flank pain in clinic and 5 cases were defined due to hematuria. All diagnoses were established depending on the evidence of bilateral renal masses on ultrasound and CT imagings before surgery. Intravenous urography (IVU) was adopted in 12 cases, in which 10 were identified with the compression of unilateral or bilateral renal pelvis or calyx. MRI demonstrated the bilateral renal masses in all 14 patients. Cancer embolus in the vena cava was detected in one patient and megatherium lymph nodes in the retroperitoneum were found in another patient. None of patients showed the signs of distant metastasis. According to the 2009 UICC/AJCC TNM staging system, 44 (68.8%) tumors were classified at T1aN0M0, 13 (20.3%) tumors were at T1bN0M0, 5 (7.8%) tumors were at T2N0M0, 1 (1.6%) tumor was at T3bN0M0, and other 1 (1.6%) tumor was at T2N1M0 (Table 1).

**Table 1:** Baseline demographic and clinical characteristics of the BSSRCC patients

Characteristics	Number(percentage), *N* = 32	
Gender		
Male	28 (87.5%)	
Female	4 (12.5%)	
Age (years)		
≥50	21 (65.6%)	
<50	11 (34.4%)	
Operation stage		
One-stage	8 (25.0%)	
Two-stage	24 (75.0%)	
TNM stage	Right side, *N* = 32	Left side, *N* = 32
T_1a_N_0_M_0_	21 (65.6%)	23 (71.8%)
T_1b_N_0_M_0_	9 (28.1%)	4 (12.5%)
T_2a_N_0_M_0_	1 (3.1%)	1 (3.1%)
T_2b_N_0_M_0_	0	3 (9.4%)
T_3b_N_0_M_0_	1 (3.1%)	0
T_2_N_1_M_0_	0	1 (3.1%)
Operation Sequence	*N* = 32	*N* = 32
First	12 (37.5%)	20 (62.5%)
Second	20 (62.5%)	12 (37.5%)
Operation methods	*N* = 32	*N* = 32
NSS	24 (75.0%)	23 (71.9%)
RN	8 (25.0%)	9 (28.1%)
ZS score	*N* = 24	*N* = 23
Low risk	5 (20.8%)	6 (26.1%)
Moderate	10 (41.6%)	8 (33.3%)
High risk	9 (37.5%)	9 (39.1%)

All tumors were stratified into three complexity levels according to Zhongshan score (ZS score) [9]. Low-risk tumors were scored between 3~4, whereas moderate tumors were scored between 5~7, and high-risk tumors were scored ≥8.

### Preoperative work-up

The preoperative work-up consists of medical history, physical examinations, and routine laboratory tests. Concentrations of serum creatinine were recorded before and after operation consecutively at an interval of 3–6 months. Estimated Glomerular filtration rate (GFR) was calculated using the modification of diet in renal disease (MDRD) formula. Postoperative assessments during follow-up period included abdominal ultrasonography and chest x-ray examinations at the interval of each 3 months in the first 2 years and at each 6 months afterward.

### Treatment

One-stage bilateral surgery was performed in eight patients, of which six cases were carried out bilateral NSS and two cases were taken RN for one side first and NSS for the opposite side thereafter. Two-stage operations were performed in other 24 cases, of which 5 cases were adopted NSS first and RN for the opposite side 4–8 weeks later; 10 cases were performed RN first and then NSS for the opposite side 4–8 weeks later; 9 cases were conducted NSS in both sides within 4–8 weeks. For one patient with tumor embolus in vena cava, an intravenous stent was placed and a filter was positioned at inferior vena cava before operation to prevent the embolus occlusion, followed by ipsilateral RN and contralateral NSS 7 weeks later. The characteristics of tumor size and the therapeutic options are summarized in Table 1. Complications were evaluated using the Clavien scale [10].

### Surgical strategy for BSSRCC

#### Selection of surgical stage

One-stage bilateral surgery (OBS): OBS is considered when physical status of the patient is fitted (ECOG PS = 0) while being without any comorbidity.

Two stages surgery (TSS): TSS is selected for the patient with large size tumor or other complications, such as cancer embolus in the vena cava. Patients with ECOG PS > 0 are also enrolled in this group.

#### Surgical sequence

**Fig. 1:**
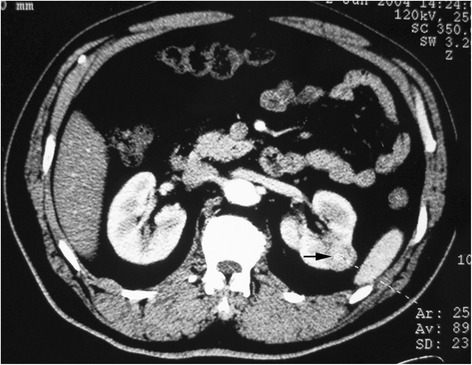
Bilateral NSS: larger tumor on the left side was dissected first

**Fig. 2:**
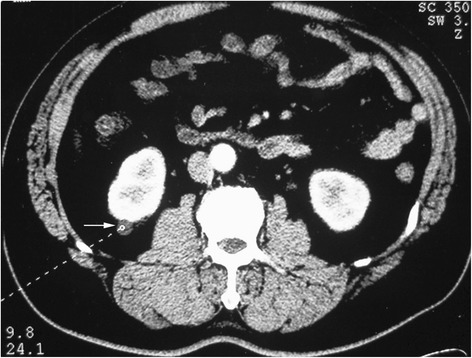
Bilateral NSS: larger tumor on the left side was dissected first (Figs. 1 and 2 were of the same patient)

**Fig. 3:**
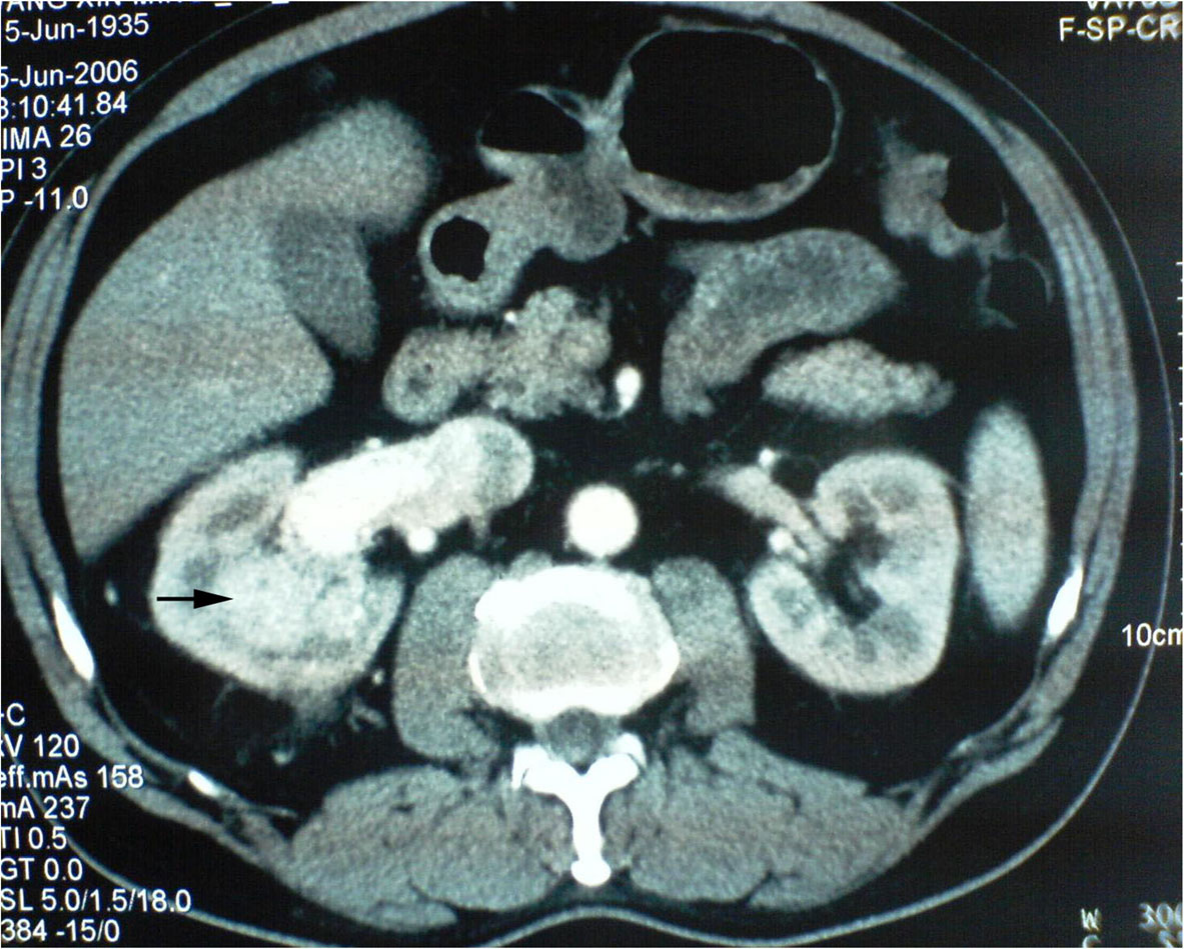
RN on the right side first and NSS on the left thereafter at second staged surgery

**Fig. 4:**
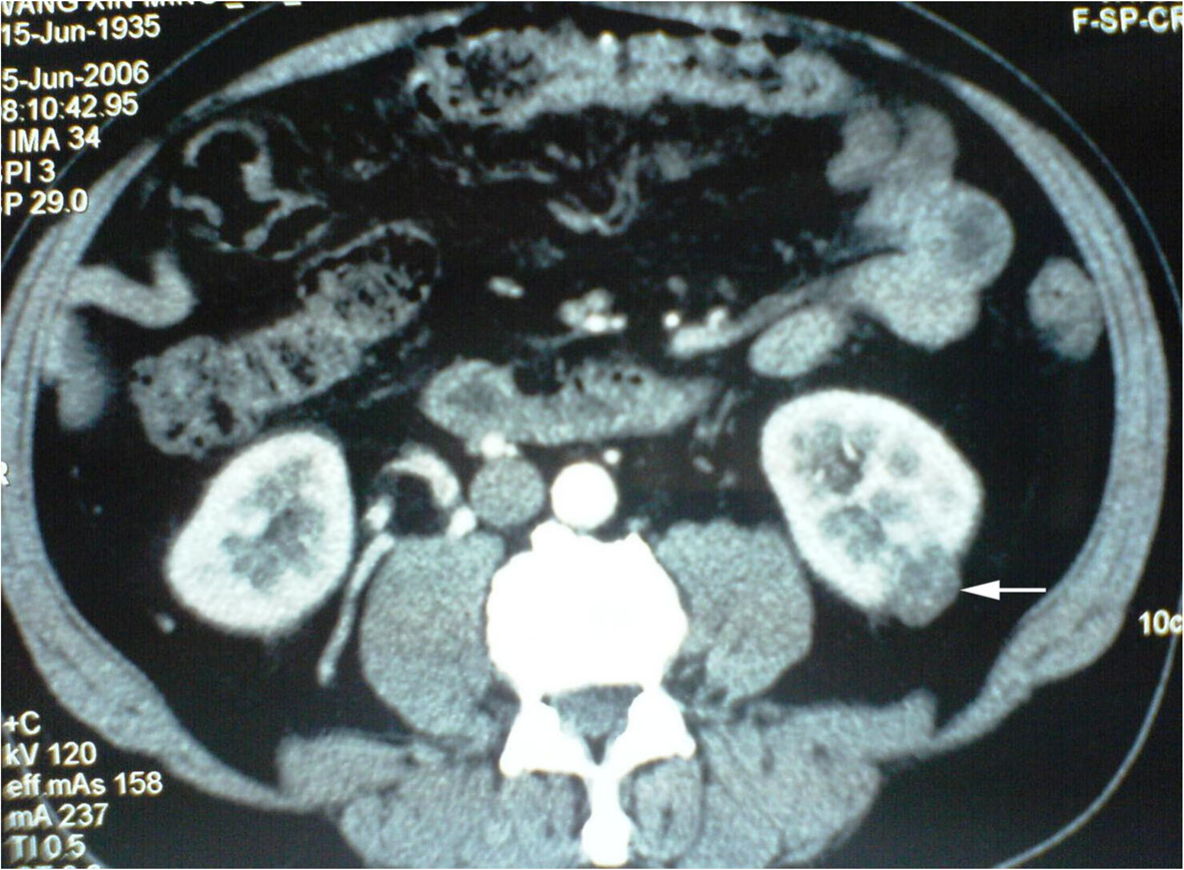
RN on the right side first and NSS on the left thereafter at second staged surgery (Figs. 3 and 4 were of the same patient)

**Fig. 5:**
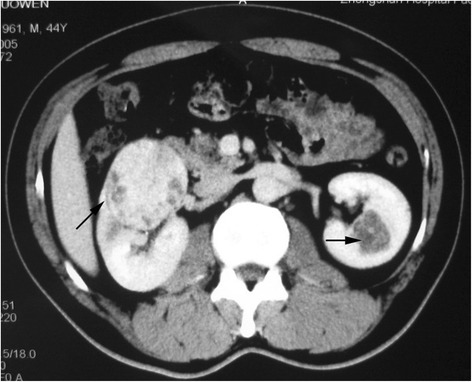
NSS on the left side first and RN on the right side at second staged surgey

For BSSRCC patients intending staged surgery, the choosing of side to be operated initially and the surgical approach should be meticulously decided based on the tumor location and size at both the kidneys. For the bilateral NSS, first-stage surgery should be conducted on the side with higher ZS score tumors (Figs. 1 and 2). For the patients determined to do radical and partial resection separately at both sides one after another, the first-stage radical surgery should be operated on the side with larger tumor as well, if the partial resection to low-moderate risk tumors in the other side were easy to perform expectedly (Figs. 3 and 4). However, if tumors were assessed at high-risk level on the one side which were planned to do partial resection, the NSS procedure should be operated earlier than contralateral RN (Fig. 5). If NSS was unsuccessful and required to switch to RN, further preparations including appropriate hemodialysis were necessary prior to the second-stage operation of contralateral RN (Table 2).

**Table 2:** Surgical sequence of the BSSRCC based on ZS score

	First step	Second step
Bilateral NSS	Higher risk side	Lower risk side
RN+NSS	RN	NSS (low-moderate risk)
	NSS (high risk)	RN

The same criteria should be met in one-stage bilateral surgery in management of surgical schedule.

## Results

A total of 56 operations were productively completed in all 32 patients (average age 54.7 years, range 31 to 77). Surgical patterns of retroperitoneal open partial nephrectomy (OPN) and transperitoneal open/laparoscopic radical nephrectomy were selectively applied. Mean operation time of OBS and TSS were 263 ± 50 min (range 200–320) and 154 ± 42 min (range105–180), mean hospitalization time of OBS and TSS were 11.4 ± 1.9 days (range 8–15) and 7.4 ± 1.3 days (range 6–9), respectively. The warm ischemia time in NSS was 26 ± 7 min (range 16–45). Pathological examination of 64 neoplasms samples revealed that 57 samples were clear cell carcinoma, 5 were papillary carcinoma, and 2 were chromophobe carcinoma.

After bilateral NSS, one patient appeared pseudoaneurysm of renal arteriole, accompanied with gross hematuria and hypovolemic shock. The patient then received the treatment of percutaneous high-selective renal arterial embolization (Clavien grade 3). Twenty-eight patients were followed up 6–138 months (median 89 months) except four patients were lost contact. The renal function was maintained in normal range in 23 patients. Increased creatinine (125–187 μmol/L) level and reduced eGFR were happened in five patients, who were treated with NSS on the one side and contralateral RN without hemodialysis. Lung metastasis was found in one patient after 14 months of RN plus contralateral NSS operation and died 25 months later. Tumor recurrence at the left nephridial pit was seen in one patient conducted with NSS on the both sides. The patient was treated with salvage radiotherapy and died 38 months later after surgery. One patient with RN and contralateral NSS died from cerebrovascular accident 6 months after surgery (Table 3).

**Table 3:** Results of following up of the BSSRCC patients

Characteristics	Number(percentage)
Lost in follow-up	4 (12.5%)
Complications	
Clavien I~II	5 (17.9%)
Clavien III	1 (3.6%)
CRI	5 (17.9%)
Hemodialysis	0
Survival	25 (89.3%)
Metastasis/recurrence	1/1 (3.6%/3.6%)

## Discussion

Although BSSRCC is an uncommon disease with lower incidence [11], the management is much more difficult and complicated than that of metachronous RCC. Accordingly, the selection of surgical approach and the schedule of operation sequence require careful evaluation of the disease and the general condition of patients.

### Surgical criteria

#### Selection of operation pattern in our study

1. Bilateral NSS: The location and size of the tumor in both sides meet the indication for NSS (Fig. 6). In present series, 15 patients were implemented bilateral NSS.

**Fig. 6:**
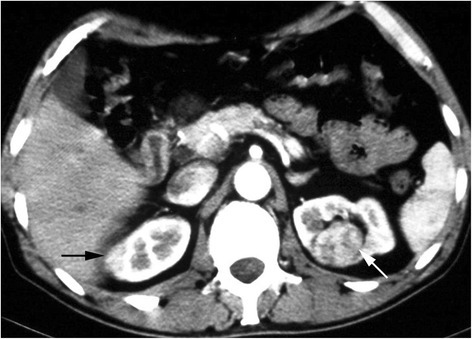
Left central renal tumor was partially resected
2. Lateral NSS on the one side and RN on the contralateral side. In general, it was applied for patients with small tumor on the one side which meet the indication for NSS and with relatively larger tumor on the other side which required RN. Seventeen patients in the cohort received surgery in this manner (Fig. 7). Four patients showed the elevation of creatinine levels after operation because of inadequate preservation of renal parenchyma. It indicated the renal parenchyma should be conserved as much as possible in NSS to avoid postoperative dialysis.

**Fig. 7:**
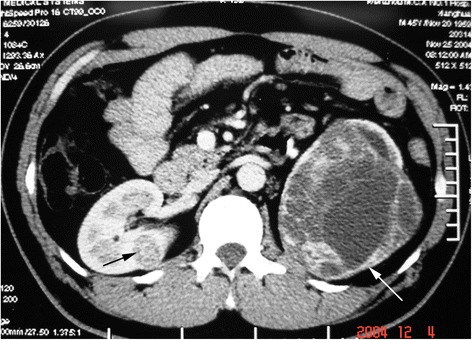
NSS on the right side and RN on the left
3. Bilateral RN. It is applied in patients with bilateral large tumors which are unsuitable for NSS. Due to the complete loss of renal function after surgery, kidney replacements are necessary to maintain the life of patients. Therefore, the selection of this surgical manner should be particularly cautious. None of our patient has taken this surgical manner.

### Surgery sequence

The individual therapeutic protocol for each patient should be created only through appropriately weighing the advantages and disadvantages of different surgical sequences, then make the selection of either one-stage or two-stage surgery manner and which side is the first for surgery. The essential principle is which manner can provide the optimal effects of thoroughly eliminating the tumor cells as early as possible. One-stage bilateral surgery may be the effective therapeutic method for BSSRCC based on the criteria in case of physical status of the patient allowed. However, the elderly patients or those with vital organ dysfunction owing to complicated status of operation either in one or two sides is better to take staged surgery after 1–2 months of the first-stage surgery done. The initial operation on one side is a pivotal procedure in staged surgery. We should focus on the high risk tumor of BSSRCC following the above criteria. But it is not always the case. Maximal preservation of renal function should be taken into account at the same time, especially in the case of NSS with contralateral RN. Consequently, we describe the therapeutic methods in combination of renal function preservation and tumor removal in making our surgical criteria.

In summary, both preservation of the renal parenchyma and tumor eradication should be considered simultaneously in management of BSSRCC. Before formulating an individual therapeutic regime, the patient’s performance status, tumor size, and location should be evaluated carefully [12] according to the ZS score. The renal parenchyma should be preserved as much as possible during the operation to prevent hemodialysis postoperatively, but complete tumor removal is the most important target for the treatment of patient.

**Fig. 8:**
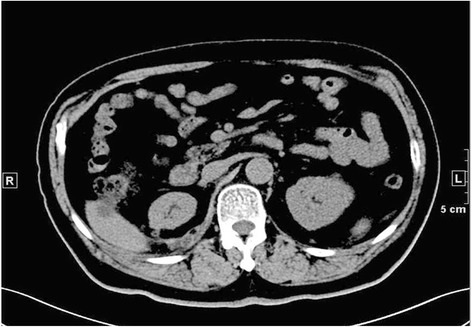
One year after bilateral NSS with normal renal function and no tumor recurrence could be found

Our newly established surgical criteria for BSSRCC, using our novel and simple score system, have demonstrated encouraging and satisfactory outcomes (Fig. 8). It is feasible and safe in clinical application. Since BSSRCC is a rare disease [13, 14], the longer follow- up period, more patient enrolment, and novel anatomic classification systems [15] are needed to further investigate the beneficial effects of different surgical approaches for the patients.

## Conclusions

We established our promising surgical criteria for BSSRCC based on the ZS score.

Both preservation of renal parenchyma and tumor eradication should be considered in BSSRCC. Before formulating a therapeutic regime, the patient’s performance status, tumor size, and location should be evaluated. Renal parenchyma should be preserved as much as possible during the operation, but complete tumor removal is more important.

Longer follow-up and more patients enrolment are required.

## Abbreviations

BSSRCC: Bilateral synchronous sporadic renal cell carcinoma
CRI: Chronic renal insufficiency
ECOG PS: Eastern cooperative oncology group, performance status
GFR: Glomerular filtration rate
IVU: Intravenous urography
NSS: Nephron-sparing surgery
OBS: One-stage bilateral surgery
OPN: Open partial nephrectomy
OS: Overall survival
RN: Radical nephrectomy
TSS: Two stages surgery
ZS score: Zhongshan score
